# Supplementary material for: Psychometric properties of the Brazilian-Portuguese Flow State Scale Short (FSS-BR-S)
Source: PLoS One. 2024 Feb 1;19(2):e0286639. doi: 10.1371/journal.pone.0286639 (PMC10833536; doi:10.1371/journal.pone.0286639)
Supplement: S1 Appendix — Portuguese-Brazilian Long Version of the Flow-State Scale 2. (PDF) [file pone.0286639.s001.pdf]

S1 Appendix. Portuguese-Brazilian Long Version of the Flow-State Scale 2 (FSS-BR)

Por favor, responda às seguintes questões com relação à sua experiência na atividade que acabou de finalizar. Não há respostas corretas ou erradas. Pense sobre sua concordância em cada uma das sentenças apresentadas e então marque o número que melhor representa sua concordância.

[EN: *Please answer the following questions regarding your experience in the activity you just completed. There are no right or wrong answers. Think about your agreement degree with each of the sentences, and then mark the number that best represents your agreement*.]

Durante: ____

Q1: Fui desafiado, mas acredito que minhas habilidades me permitiriam enfrentar o desafio

◉ Discordo fortemente ○ Discordo ○ Nem concordo nem discordo ○ Concordo ○ Concordo fortemente

Q2: Fiz as coisas corretamente sem pensar em como fazer

◉ Discordo fortemente ○ Discordo ○ Nem concordo nem discordo ○ Concordo ○ Concordo fortemente

Q3: Sabia claramente o que queria fazer

◉ Discordo fortemente ○ Discordo ○ Nem concordo nem discordo ○ Concordo ○ Concordo fortemente

Q4: Estava muito claro para mim como eu estava me saindo na atividade

◉ Discordo fortemente ○ Discordo ○ Nem concordo nem discordo ○ Concordo ○ Concordo fortemente

Q5: Minha atenção estava focada inteiramente no que eu estava fazendo

◉ Discordo fortemente ○ Discordo ○ Nem concordo nem discordo ○ Concordo ○ Concordo fortemente

Q7: Não estava preocupado com o que os outros podiam estar pensando de mim

◉ Discordo fortemente ○ Discordo ○ Nem concordo nem discordo ○ Concordo ○ Concordo fortemente

Q8: O tempo pareceu estar alterado (mais lento ou mais acelerado)

◉ Discordo fortemente ○ Discordo ○ Nem concordo nem discordo ○ Concordo ○ Concordo fortemente

Q9: Realmente curti a experiência da atividade que estava fazendo

◉ Discordo fortemente ○ Discordo ○ Nem concordo nem discordo ○ Concordo ○ Concordo fortemente

Q10: Minhas habilidades combinavam com o desafio da atividade que estava fazendo

◉ Discordo fortemente ○ Discordo ○ Nem concordo nem discordo ○ Concordo ○ Concordo fortemente

Q11: As coisas pareciam estar acontecendo automaticamente

◉ Discordo fortemente ○ Discordo ○ Nem concordo nem discordo ○ Concordo ○ Concordo fortemente

Q12: Tive uma clara noção do que queria fazer

◉ Discordo fortemente ○ Discordo ○ Nem concordo nem discordo ○ Concordo ○ Concordo fortemente

Q13: Estava consciente do quão bem eu estava fazendo

Discordo fortemente Discordo Nem concordo nem discordo Concordo Concordo fortemente

Q14: Não me esforcei para manter a minha mente no que estava acontecendo

Discordo fortemente Discordo Nem concordo nem discordo Concordo Concordo fortemente

Q15: Senti que poderia controlar o que estava fazendo

Discordo fortemente Discordo Nem concordo nem discordo Concordo Concordo fortemente

Q16: Não estava preocupado sobre como os outros podem ter me avaliado

Discordo fortemente Discordo Nem concordo nem discordo Concordo Concordo fortemente

Q17: A forma como o tempo passou parecia ser diferente do normal

Discordo fortemente Discordo Nem concordo nem discordo Concordo Concordo fortemente

Q18: Amei a sensação que estava experimentando, e quero ter esse sentimento novamente

Discordo fortemente Discordo Nem concordo nem discordo Concordo Concordo fortemente

Q19: Senti que era competente o suficiente para atender às demandas da situação

Discordo fortemente Discordo Nem concordo nem discordo Concordo Concordo fortemente

Q20: Fiz coisas automaticamente, sem pensar muito

Discordo fortemente Discordo Nem concordo nem discordo Concordo Concordo fortemente

Q21: Eu sabia o que queria alcançar

Discordo fortemente Discordo Nem concordo nem discordo Concordo Concordo fortemente

Q22: Tive uma boa ideia sobre como estava me saindo na atividade quando estava envolvido nela

Discordo fortemente Discordo Nem concordo nem discordo Concordo Concordo fortemente

Q23: Tive total concentração

Discordo fortemente Discordo Nem concordo nem discordo Concordo Concordo fortemente

Q24: Tive uma sensação de total controle sobre o que estava fazendo

Discordo fortemente Discordo Nem concordo nem discordo Concordo Concordo fortemente

Q25: Não estava preocupado com a forma como estava me apresentando

Discordo fortemente Discordo Nem concordo nem discordo Concordo Concordo fortemente

Q26: Pareceu que o tempo passou rapidamente

Discordo fortemente Discordo Nem concordo nem discordo Concordo Concordo fortemente

Q27: A experiência me deixou com uma ótima sensação

◉ Discordo fortemente ○ Discordo ○ Nem concordo nem discordo ○ Concordo ○ Concordo fortemente

Q28: O desafio e minhas habilidades estavam em um nível igualmente alto

◉ Discordo fortemente ○ Discordo ○ Nem concordo nem discordo ○ Concordo ○ Concordo fortemente

Q29: Fiz coisas automaticamente sem ter que pensar

◉ Discordo fortemente ○ Discordo ○ Nem concordo nem discordo ○ Concordo ○ Concordo fortemente

Q30: Meus objetivos estavam claramente definidos

◉ Discordo fortemente ○ Discordo ○ Nem concordo nem discordo ○ Concordo ○ Concordo fortemente

Q31: Eu poderia dizer, pela forma como as coisas estavam progredindo, quão bem eu estava indo

◉ Discordo fortemente ○ Discordo ○ Nem concordo nem discordo ○ Concordo ○ Concordo fortemente

Q32: Estava completamente focado na tarefa em questão

◉ Discordo fortemente ○ Discordo ○ Nem concordo nem discordo ○ Concordo ○ Concordo fortemente

Q33: Eu me senti no total controle das minhas ações

◉ Discordo fortemente ○ Discordo ○ Nem concordo nem discordo ○ Concordo ○ Concordo fortemente

Q34: Eu não estava preocupado com o que os outros podiam estar pensando de mim

◉ Discordo fortemente ○ Discordo ○ Nem concordo nem discordo ○ Concordo ○ Concordo fortemente

Q35: Perdi a noção do tempo

◉ Discordo fortemente ○ Discordo ○ Nem concordo nem discordo ○ Concordo ○ Concordo fortemente

Q36: Achei a experiência extremamente recompensadora

◉ Discordo fortemente ○ Discordo ○ Nem concordo nem discordo ○ Concordo ○ Concordo fortemente
