# Supplementary material for: Psychometric properties of the Brazilian-Portuguese Flow State Scale Short (FSS-BR-S)
Source: PLoS One. 2024 Feb 1;19(2):e0286639. doi: 10.1371/journal.pone.0286639 (PMC10833536; doi:10.1371/journal.pone.0286639)
Supplement: S2 Appendix — Portuguese-Brazilian Short Version of the Flow-State Scale 2. (PDF) [file pone.0286639.s002.pdf]

S2 Appendix. Short version of the Flow State Scale (FSS-Short BR)

Por favor, responda às seguintes questões com relação à sua experiência na atividade que acabou de finalizar. Não há respostas corretas ou erradas. Pense sobre sua concordância em cada uma das sentenças apresentadas e então marque o número que melhor representa sua concordância.

[EN: *Please answer the following questions regarding your experience in the activity you just completed. There are no right or wrong answers. Think about your agreement degree with each of the sentences, and then mark the number that best represents your agreement*.]

Durante: ____

Q4: Estava muito claro para mim como eu estava me saindo na atividade

◉ Discordo fortemente ○ Discordo ○ Nem concordo nem discordo ○ Concordo ○ Concordo fortemente

Q7: Não estava preocupado com o que os outros podiam estar pensando de mim

◉ Discordo fortemente ○ Discordo ○ Nem concordo nem discordo ○ Concordo ○ Concordo fortemente

Q10: Minhas habilidades combinavam com o desafio da atividade que estava fazendo

◉ Discordo fortemente ○ Discordo ○ Nem concordo nem discordo ○ Concordo ○ Concordo fortemente

Q11: As coisas pareciam estar acontecendo automaticamente.

◉ Discordo fortemente ○ Discordo ○ Nem concordo nem discordo ○ Concordo ○ Concordo fortemente

Q23: Tive total concentração

◉ Discordo fortemente ○ Discordo ○ Nem concordo nem discordo ○ Concordo ○ Concordo fortemente

Q24: Tive uma sensação de total controle sobre o que estava fazendo

◉ Discordo fortemente ○ Discordo ○ Nem concordo nem discordo ○ Concordo ○ Concordo fortemente

Q26: Pareceu que o tempo passou rapidamente

◉ Discordo fortemente ○ Discordo ○ Nem concordo nem discordo ○ Concordo ○ Concordo fortemente

Q27: A experiência me deixou com uma ótima sensação

◉ Discordo fortemente ○ Discordo ○ Nem concordo nem discordo ○ Concordo ○ Concordo fortemente

Q30: Meus objetivos estavam claramente definidos

◉ Discordo fortemente ○ Discordo ○ Nem concordo nem discordo ○ Concordo ○ Concordo fortemente
