# Supplementary material for: Psychometric properties of the Brazilian-Portuguese Flow State Scale Short (FSS-BR-S)
Source: PLoS One. 2024 Feb 1;19(2):e0286639. doi: 10.1371/journal.pone.0286639 (PMC10833536; doi:10.1371/journal.pone.0286639)
Supplement: Attachment — Submitted filename: Response to reviewers.pdf [file pone.0286639.s004.pdf]

Responding to Reviewer Comments

Brazilian-Portuguese Flow State Scale 2 (FSS-BR): Psychometric properties

Dear reviewers of PLOS ONE, we would like to thank you for your insightful comments and suggestions; each one has been carefully analyzed and considered in the new version of the paper. We also indicate in this letter the texts that were changed or included in the manuscript to answer all comments. We hope that all these changes fulfill the requirements to make the manuscript for its publication.

Looking forward to hearing from you.

Reviewer 1

Comment 1: *I commend the authors on attempts to develop a measure of flow for the specified population. The sample is a reasonable size for this type of analysis. However, I have some issues from a methodological perspective as it does not appear as to which version of the flow scales is being used in the study. At times, the FSS is stated, while at others it is the FSS-2.*

Answer: Thanks for your comment, we adjusted the text to be more clear about the use of the terms: FSS-2 and FSS-BR. We used the term FFS-2 to refer to the English version of the instrument to measure the flow state, and we used the term of FSS-BR to refer to the Portuguese-Brazilian version.

Comment 2: *From a theoretical perspective, the study places a lot of faith in the nine dimensions framework, but this framework, while widely used, has been criticised and one would expect more engagement with such literature to justify the theoretical approach adopted here. In addition, the flow channel model stated is somewhat dated; indeed, in 1988, Csikszentmihalyi and Csikszentmihalyi proposed an alternative to this.*

Answer: Thanks for your comment. Indeed, there are a lot of criticisms of the 09-factor model of the Csikszentmihalyi' flow, but our objective was not to provide evidence that supports this theoretical approach. Our main contribution was the construction and validation of an instrument to measure the flow

state but based on a single dimension with only nine items. To achieve this goal, we started adapting and validating a translated version of an instrument of 09-factors and 36 itens. Instrument which was based on the original version of the FSS-2, and translated to Brazilian-Portuguese, and developed to be applied to general activities.

Comment 3: *Finally, there is a room for the standard of written English to be improved throughout.*

Answer: Dear reviewer, thank you for your comment and the written English was improved.

Reviewer 2

Comment 1 - Major suggestions: *First, as you state, one goal of validating indices in other contexts is to allow comparison of results; given that goal, I'm surprised that you so readily suggest modified versions of the FSS-2 (i.e., 28 items instead of 36). With the data you've presented, I'm not confident that the 28-item models do fit all that much better than the 36-item models. As I note below, I don't agree that the 28-item model is nested within the 36-item model. If the two models either can't be compared directly (which I don't believe is true) or are found to be – through comparison – fairly similar, my strong suggestion would be for you to champion the model that is already published so that your and future results would be more easily compared to previous work. Importantly, you have presented data on the original 36-item model, so that is a great first step, but your suggestion that future work use the 28-item model seems under-supported and perhaps overly hasty. To put this more simply, my primary question is whether you have successfully adapted Jackson and colleagues FSS-2 to a Brazilian context?*

Answer: Indeed, we successfully adapted and validated the original structure of the English version of the FSS-2 for Brazilian-Portuguese language. Our proposed 28-item model is similar to the multi-correlated nine-factor model with 36-item. There is not really a difference among the two models, thereby we updated the article removing the assessment of the 28-item model, and evaluating only the two models widely evaluated in the literature: the nine-factor multi-correlated model (Jackson, S. A. et al., 2008); and the second-order nine-factor model (Riva, E.F. et al., 2017).

- Jackson, S. A., Martin, A. J., & Eklund, R. C. (2008). Long and short measures of flow: The construct validity of the FSS-2, DFS-2, and new brief counterparts. *Journal of Sport and Exercise Psychology*, *30*(5), 561-587.
- Riva, E. F., Riva, G., Talò, C., Boffi, M., Rainisio, N., Pola, L., ... & Inghilleri, P. (2017). Measuring dispositional flow: validity and reliability of the dispositional flow state scale 2, Italian version. *PloS one*, *12*(9), e0182201.

Comment 2 - Major suggestions: *As you rightfully point out, your sample is not ideal; this is certainly a limitation of your study, but it needs to be addressed more thoroughly in locations outside of the limitations section (e.g., in the methods section).*

Answer: Thanks, we added a paragraph in the subsection "data analysis procedure" (inside methods section) indicating the limitation of our sample as suggested by you.

Comment 3 - Minor suggestions: *I would move your recruitment section above your participants section to let readers because that information bears on your reader's understanding of those participants.*

Answer: thank you and we followed your suggestion and moved the recruitment section above the participants section.

Comment 4 - Minor suggestions: *Regarding demographics reporting: "less than 18 years old" is a wide and varied group of respondents. I understand it may not be possible to thoroughly describe this (large) group of your respondents, but some acknowledgement or reporting of the characteristics of that group would be helpful, particularly considering in many contexts (e.g., many studies conducted in the U.S.), people under 18 are not able to complete surveys without consent of a parent or guardian.*

Answer: All respondents, including parents of minors, all of them agreed and signed a Free Prior and Informed Consent (FPIC). All participants under 18 y/o were at least adolescents

(their ages were between 15 y/o and less than 18 y/o). In this sense, we clarified this information in the article.

__Comment 5 - Minor suggestions:__ *Terms like “yellow” and “mongoloid” can be offensive in some contexts. Was this the exact language in your questionnaire? If not, you might consider more neutral language (such as just “Asian”).*

Answer: Thanks for your observation. We change the term as you suggested.

__Comment 6 - Minor suggestions:__ *It seems that many of your respondents failed to answer a number of demographic questions – upwards of 40% for some questions. Do you believe this is a systematic source of missing data? Can you speak to this concern, either in a response to reviewers and/or in the manuscript?*

Answer: Responses were gathered after people participated in different activities. One of these activities corresponds to “solving logic problems in a gamified environment to measure aggressiveness.” Due to the sensibility of this activity, there was not gathered demographic information after this activity. Thereby, we explicitly included this information in the article.

__Comment 7 - Minor suggestions:__ *How were the flow-state activities identified by respondents? Were they asked an open-ended question that allowed them to identify any activity, or did you provide them with a list of activities to select from? This is not clear but seems crucial to the quality of your data. You should also identify – to the extent possible – how distant the flow state was; did participants experience the flow state recently, months ago, etc.?*

Answer: The activities performed by the respondents were explicitly indicated in an open-ended question immediately after the respondents participated in these activities. We added this information in the article.

__Comment 8 - Minor suggestions:__ *Table 1 should more clearly identify what the p-value column refers to – I believe the Shapiro-Wilk test that you reference in text.*

Answer: Thanks, the heads of Table 1 are now more detailed in the article text.

Comment 9 - Minor suggestions: *This statement confuses me: "To compare whether or not the models are nested…" Your models are nested if you can obtain one model through modification to the others. This is a logical, rather than statistical argument. In your case, I don't believe your 28-item models can be nested within your 36-item models, since you are eliminating items – not just paths. The ANOVA test that you reference could presumably tell you if a nested (simpler) model fits better than its parent (more complex) model, but only in the case of a nested model, which – again, I don't believe you have. My recommendation would be to report Akaike Information Criteria (AIC) or Bayesian Information Criteria (BIC) for each model, since these metrics are comparable across non-nested models. To be clear: I don't disagree that the 28-item models might fit better (my assumption is that this is actually the case), but I don't believe you've provided the right evidence to support that conclusion.*

Answer: Thanks for your observations, we calculated AIC and BIC indexes (using the Maximum likelihood estimator), and we included these indexes in Tables 2 and 8 in which we presented fit indexes of the CFAs. As we removed the 28-item models in the CFAs, we also removed the ANOVA test results.

Comment 10 - Minor suggestions: *I don't know that I would consider your results for the difficulty of the items to be a good thing. All of your items were answered as if they were relatively easy (i.e., had negative bx), but I don't know that flow is necessarily an "easy" state to get into. Again, some additional context on how respondents were tasked with identifying their flow experience would be helpful here; for example, if the experience was relatively recent (vs. distant), I think answering a flow questionnaire would be considerably easier. I think, ideally, you would have items with a variety of difficulty – some easy and some hard, but all of these seem to be easy for your sample.*

Answer: To improve the readability of the text, and don't cause confusion, we removed the scale names indicated by Pasquali (2020). We also added information about the meaning of the difficulty parameters in terms of the flow state when their average (bx) were presented.

- Pasquali, L. (2020). *TRI–Teoria de resposta ao item: Teoria, procedimentos e aplicações*. Editora Appris.

Comment 11 - Minor suggestions: *I don't think it should reasonably take a person a full minute to answer each question in the flow state scale (or in many other indices), so I'd probably drop that line of reasoning. Your point that a shorter index may be preferable is still valid, but that overestimate of the time it takes to answer a question detracts from your message.*

Answer: Thanks, we removed the argument as you suggested.

Reviewer 3

Comment 1 - General comments: *The present study examines the factor structure of the Flow State Scale 2 and FSS-2 Short Form among Brazilian-Portuguese Adolescents. The study seems really very important, it's a well-written paper, however there are some points in the manuscript that need to be taken into consideration (theoretical background, and practical considerations)*

Answer*:* dear reviewer, thank you very much for your comment.

Comment 2 - Introduction: *The authors seem very familiar with the construction and validation procedure in various languages of the Flow State Scale 2 – Short Form.*

With fully respect the authors work, there must be clearer to the reader based on the previous research findings the rationality and the practical information of the current study. So the

purpose and hypotheses must be clearly stated, and a comprehensive rationality of the study, based on the theoretical background of the study, needs to be established.

Answer*:* Thank you for your comment and we improved the paper accordingly.

***<u>Comment 3 - Method/Results</u>**: The method used as well as the statistical analysis and results are generally OK. I don't have any comments regarding the statistical analysis which is very detailed examining the psychometric properties of the instrument.*
However, the information regarding the translation procedure of the FSS-2 into Brazilian Portuguese language is very limited. I propose the authors should add more information regarding the translation and the wording modifications on the items.

Answer: The items were directly translated from english to portuguese (forward translation) and verify their translation making the translation from portuguese to english (backwards translation), each one by independent researchers, and in two independent phases. Therefore, the wording modification between these two phases were not recorded. The modification between english and portuguese can be easily inferred compared each one the items presented in the appendix S1 Appendix and the items in the original version of FSS-2 (available in Jackson, 2010)

- Jackson, S. A., Eklund, R. C., & Martin, A. J. (2010). *The FLOW manual: The manual for the flow scales*. Mind Garden, Incorporated.

***<u>Comment 3.1 - Method/Results:</u>** Lines 173-178. This analysis is used for exploratory factor analysis, which was something that the authors didn't use.*

Answer: *CFA and EFA both assume a normal distribution and they share the same assumption.*

***<u>Comment 3.2 - Method/Results:</u>** Line 267. The ratio of the x2/df ration is high for all examined models.*

Answer: Thanks for your observation, we made a typo error, the proper word is "acceptable" instead of "good." We based the classification in the ranges defined by Hu and Bentler (1999).

- Hu, L. T., & Bentler, P. M. (1999). Cutoff criteria for fit indexes in covariance structure analysis: Conventional criteria versus new alternatives. *Structural equation modeling: a multidisciplinary journal*, *6*(1), 1-55.

Comment 3.3 - Method/Results: *Lines 268-270. The authors should clearly define the criteria of omitting the items in the 28- items model. The criteria are only statistical? In addition, should present the reasons of excluding one of the nine factors of the FSS-2 instrument. The criteria should not be only statistical, but mainly theoretical.*

Answer: We based the removal of items for the 28-itens model based on the cutoff criteria defined by Stevens (2012). In the new version of the article, we removed the 28-items model for our CFA, being only evaluating the fit indexes of two models: the original nine-multi correlated model, and the second order nine-factors model. Both models were acceptable.

- Stevens, J. P. (2012). *Applied multivariate statistics for the social sciences*. Routledge.

Comment 3.4 - Method/Results: *Line 298-304. The internal consistency and reliability of the FSS-2 factors is low.*

Answer: The consistency and reliability were acceptable with values of 0.60s.

Comment 4 - Discussion: *In general, it is a well-written research work. The authors did a good work regarding the examination of the psychometric properties of the FSS-2.*

With all my respect to the authors' scientific work, I think that it is important to explain much more clearly what the unique contribution of this study is e.g., factor structure invariance across age, exercise participation. Although it was not the research purpose of the authors in the current study however examining the convergent, concurrent, discriminant validity will provide important information regarding the "applicability" and the "practical information" of the instrument.

Answer*:* Thanks for your comment. Applicability and practical information of our validated psychometric instrument. We can't conduct MGCFA to assess the invariance of our instrument due to the limit of sampling method. We pointed out this fact, and we also included the justification in the article.

- These both instruments cannot be used for diagnosis, but they can be applied in any Brazilian context and for any activity, to have a better understanding about the phenomena of flow experience
