# Supplementary material for: Psychometric properties of the Brazilian-Portuguese Flow State Scale Short (FSS-BR-S)
Source: PLoS One. 2024 Feb 1;19(2):e0286639. doi: 10.1371/journal.pone.0286639 (PMC10833536; doi:10.1371/journal.pone.0286639)
Supplement: Attachment — Submitted filename: Response to Reviewers.pdf [file pone.0286639.s006.pdf]

Responding to Reviewer Comments

Dear Academic Editor and Reviewers of PLOS ONE, we would like to thank you for your comments; each has been carefully analyzed and considered in the new version of the paper. The most current version of the manuscript was adjusted in this direction and te hope that all these changes fulfill the requirements to make the manuscript ready for publication.

Review comments to the author:

Reviewer 1 - Comment 6: *I would like to thank the authors for making all of the recommended revisions. The manuscript is much clearer and is easier to read.*

Some minor revisions remain, mostly typos. They are as follows:
line 165--change "constrct" to "construct"
line 168--change "TRI" to "IRT"
line 177--move "(CFA)" to line 176 after the word "Analysis"
line 336--Is the range supposed to be -3, +3? Currently it states 3, +3
line 386--changes "quetions" to "questions"
line 411--change "rang" to "range"
line 456 change "this" to "these"

Once these minor revisions are made, I recommend this article for publication.
Answer:

Dear Reviewer, thanks for your observation and comments. The text of the manuscript was revised, and we removed all typos.
